# Notification that new names of prokaryotes, new combinations and new taxonomic opinions have appeared in volume 75, part 3 of the IJSEM

**DOI:** 10.1099/ijsem.0.006765

**Published:** 2025-07-01

**Authors:** Aharon Oren, Markus Göker

**Affiliations:** 1 The Institute of Life Sciences, The Hebrew University of Jerusalem, The Edmond J. Safra Campus, 9190401 Jerusalem, Israel; 2 Leibniz Institute DSMZ – German Collection of Microorganisms and Cell Cultures, Inhoffenstrasse 7B, 38124 Braunschweig, Germany

This listing of names of prokaryotes published in a previous issue of the IJSEM is provided as a service to microbiology to assist in the recognition of new names and new combinations. This procedure was proposed by the Judicial Commission [1]. The names given herein are listed according to the Rules of priority (i.e. time and date of publication of the articles on the journal’s platform and the order of valid publication of names in the original articles) [2].

**Table:** 

Order of article publication	Name/authors	Proposed as	Article no.	Page no.
1	*Niallia tiangongensis* Yuan *et al*. 2025	sp. nov.	006693	7
2	*Methanococcoides cohabitans* Li *et al*. 2025	sp. nov.	006685	7
3	*Christiangramia sediminicola* Wang *et al*. 2025	sp. nov.	006703	5
4	*Endozoicomonas lisbonensis* da Silva *et al*. 2025	sp. nov.	006696	9
5	*Dentiradicibacter* Bartsch *et al*. 2025	gen. nov.	006690	6
5	*Dentiradicibacter hellwigii* Bartsch *et al*. 2025	sp. nov.	006690	6
6	*Fructilactobacillus frigidiflavus* Pham and Gänzle 2025	sp. nov.	006726	8
6	*Fulvimarina uroteuthidis* Moon *et al*. 2025	sp. nov.	006683	7
7	*Pseudarthrobacter naphthalenicus* Liu *et al*. 2025	sp. nov.	006698	15
7	*Terripilifer* Liu *et al*. 2025	gen. nov.	006698	15
7	*Terripilifer ovatus* Liu *et al*. 2025	sp. nov.	006698	15
7	*Extensimonas soli* Liu *et al*. 2025	sp. nov.	006698	16
7	*Curvibacter soli* Liu *et al*. 2025	sp. nov.	006698	16
8	*Limibacterium* Gu *et al*. 2025	gen. nov.	006702	7
8	*Limibacterium fermenti* Gu *et al*. 2025	sp. nov.	006702	7
9	*Pelomonas baiyunensis* Lu *et al*. 2025	sp. nov.	006707	8
9	*Pelomonas candidula* Lu *et al*. 2025	sp. nov.	006707	8
9	*Pelomonas dachongensis* Lu *et al*. 2025	sp. nov.	006707	9
9	*Pelomonas lactea* Lu *et al*. 2025	sp. nov.	006707	10
9	*Pelomonas margarita* Lu *et al*. 2025	sp. nov.	006707	10
9	*Pelomonas nitida* Lu *et al*. 2025	sp. nov.	006707	10
9	*Pelomonas parva* Lu *et al*. 2025	sp. nov.	006707	10
9	*Roseateles rivi* Lu *et al*. 2025	sp. nov.	006707	11
9	*Kinneretia aquae* (Woo *et al*. 2024) Lu *et al*. 2025	comb. nov. [basonym: *Roseateles aquae* Woo *et al*. 2024]	006707	11
9	*Kinneretia subflava* (Woo *et al*. 2024) Lu *et al*. 2025	comb. nov. [basonym: *Roseateles subflavus* Woo *et al*. 2024]	006707	11
9	*Kinneretia violae* (Woo *et al*. 2024) Lu *et al*. 2025	comb. nov. [basonym: *Roseateles violae* Woo *et al*. 2024]	006707	11
9	*Kinneretia sediminis* (Jung *et al*. 2024) Lu *et al*. 2025	comb. nov. [basonym: *Paucibacter sediminis* Jung *et al*. 2024]	006707	12
9	*Pelomonas caseinilytica* Lu *et al*. 2025	sp. nov.	006707	12
9	*Pelomonas cellulosilytica* Lu *et al*. 2025	sp. nov.	006707	12
10	*Xanthomonas bundabergensis* McKnight *et al*. 2025	sp. nov.	006686	8
10	*Xanthomonas medicagonis* McKnight *et al*. 2025	sp. nov.	006686	9
10	*Xanthomonas tesorieronis* McKnight *et al*. 2025	sp. nov.	006686	9
11	*Rhodococcus parequi* Vazquez-Boland *et al*. 2025	sp. nov.	006679	7
12	*Microbacterium candidum* Wen *et al*. 2025	sp. nov.	006715	7
12	*Microbacterium capsulatum* Wen *et al*. 2025	sp. nov.	006715	8
13	*Novosphingobium rhizovicinum* Sun *et al*. 2025	sp. nov.	006710	9
13	*Novosphingobium kalidii* Sun *et al*. 2025	sp. nov.	006710	9
14	*Paenibacillus campi* Yao *et al*. 2025	sp. nov.	006708	6
15	*Ureaplasma ceti* Segawa *et al*. 2025	sp. nov.	006712	8
16	*Streptococcus mitis* subsp. *mitis* (Andrewes and Horder 1906) Kilian *et al*. 2025*	comb. nov. [basonym: *Streptococcus mitis* Andrewes and Horder 1906 (Approved Lists 1980)]	006704	16
16	*Streptococcus mitis* subsp. *carlssonii* Kilian *et al*. 2025†	subsp. nov.	006704	17
17	*Cupriavidus phytorum* Chávez-Ramírez *et al*. 2025	sp. nov.	006709	9
18	*Sulfitobacter sediminis* Jayan *et al*. 2025	sp. nov.‡	006705	8
19	*Streptomyces okerensis* Nouioui *et al*. 2025	sp. nov.	006716	8
19	*Streptomyces stoeckheimensis* Nouioui *et al*. 2025	sp. nov.	006716	8
20	*Qipengyuania profunda* Lee *et al*. 2025	sp. nov.	006713	9
21	*Streptomyces solicamelliae* Zheng *et al*. 2025	sp. nov.	006714	9
21	*Streptomyces solicathayae* Zheng *et al*. 2025	sp. nov.	006714	9
22	*Kitasatospora camelliae* Li *et al*. 2025	sp. nov.	006717	6
23	*Mycobacterium servetii* Tristancho-Baró *et al*. 2025	sp. nov.	006727	9
24	*Fructilactobacillus frigidiflavus* Pham and Gänzle 2025	sp. nov.	006726	8
24	*Levilactobacillus lettrarii* Pham and Gänzle 2025	sp. nov.	006726	8
25	*Buchananella felis* Feyer *et al*. 2025	sp. nov.	006723	6
26	*Roseovarius conchicola* Ha *et al*. 2025	sp. nov.	006725	6
26	*Aliiroseovarius conchicola* Ha *et al*. 2025	sp. nov.	006725	9
27	*Marivivens marinus* corrig. Kang *et al*. 2025§	sp. nov.	006735	7
28	*Psychrobacter saeujeotis* Kim *et al*. 2025	sp. nov.	006734	8
29	*Brevibacterium koreense* Nam *et al*. 2025	sp. nov.	006722	5
30	*Dennerimonas* Sbissi *et al*. 2025	gen. nov.	006733	6
30	*Dennerimonas populi* (Li *et al*. 2018) Sbissi *et al*. 2025	comb. nov. [basonym: *Aureimonas populi* Li *et al*. 2018]	006733	7
30	*Dennerimonas mangrovi* (Huang *et al*. 2021) Sbissi *et al*. 2025	comb. nov. [basonym: *Aureimonas mangrovi* Huang *et al*. 2021]	006733	7
30	*Rathsackimonas* Sbissi *et al*. 2025	gen. nov.	006733	7
30	*Rathsackimonas ureilytica* (Weon *et al*. 2007) Sbissi *et al*. 2025	comb. nov. [basonym: *Aurantimonas ureilytica* Weon *et al*. 2007]¶	006733	7
30	*Rathsackimonas phyllosphaerae* (Madhaiyan *et al*. 2013) Sbissi *et al*. 2025	comb. nov. [basonym: *Aureimonas phyllosphaerae* Madhaiyan *et al*. 2013]	006733	7
30	*Rathsackimonas jatrophae* (Madhaiyan *et al*. 2013) Sbissi *et al*. 2025	comb. nov. [basonym: *Aureimonas jatrophae* Madhaiyan *et al*. 2013]	006733	8
30	*Rathsackimonas galii* (Aydogan *et al*. 2016) Sbissi *et al*. 2025	comb. nov. [basonym: *Aureimonas galii* Aydogan *et al*. 2016]	006733	8
30	*Rathsackimonas pseudogalii* (Aydogan *et al*. 2016) Sbissi *et al*. 2025	comb. nov. [basonym: *Aureimonas pseudogalii* Aydogan *et al*. 2016]	006733	8
30	*Rathsackimonas flava* (Tuo and Yan 2019) Sbissi *et al*. 2025	comb. nov. [basonym: *Aureimonas flava* Tuo and Yan 2019]	006733	8
30	*Plantimonas* Sbissi *et al*. 2025	gen. nov.	006733	8
30	*Plantimonas endophytica* (Li *et al*. 2017) Sbissi *et al*. 2025	comb. nov. [basonym: *Aureimonas endophytica* Li *et al*. 2017]	006733	8
30	*Plantimonas psammosilenae* (Huang *et al*. 2021) Sbissi *et al*. 2025	comb. nov. [basonym: *Aureimonas psammosilenae* corrig. Huang *et al*. 2021]	006733	9
30	*Plantimonas rubiginis* (Lin *et al*. 2013) Sbissi *et al*. 2025	comb. nov. [basonym: *Aureimonas rubiginis* Lin *et al*. 2013]	006733	9
30	*Plantimonas ferruginea* (Lin *et al*. 2013) Sbissi *et al*. 2025	comb. nov. [basonym: *Aureimonas ferruginea* Lin *et al*. 2013]	006733	9
30	*Plantimonas leprariae* (Zhang *et al*. 2021) Sbissi *et al*. 2025	comb. nov. [basonym: *Aureimonas leprariae* Zhang *et al*. 2021]#	006733	9
30	*Mesocryomonas* Sbissi *et al*. 2025	gen. nov.	006733	9
30	*Mesocryomonas glaciei* (Guo *et al*. 2017) Sbissi *et al*. 2025	comb. nov. [basonym: *Aureimonas glaciei* Guo *et al*. 2017]	006733	9
30	*Jiella glaciistagni* (Cho *et al*. 2015) Sbissi *et al*. 2025	comb. nov. [basonym: *Aureimonas glaciistagni* Cho *et al*. 2015]	006733	10
31	*Mumia qirimensis* Kachor *et al*. 2025	sp. nov.	006737	5
32	*Pseudomonas gorinensis* Gonzalez *et al*. 2025	sp. nov.	006739	10

* The basonym is classified as Risk Group 2.

† The species is classified as Risk Group 2.

‡ Originally published as gen. nov., sp. nov. A corrigendum was published (DOI 10.1099/ijsem.0.006740).

§ The name *Marivivens marinum* was corrected by the List Editors to *Marivivens marinus*. The manuscript with the name *Marivivens marinum* was not reviewed by the nomenclature reviewers of the IJSEM.

¶ The authors gave as the basonym *Aureimonas ureilytica* Weon *et al*. 2007.

# The authors gave Zhang *et al*. 2020 instead *et al*. 2021.

The following taxonomic opinions (i.e. the emendation of circumscriptions or the creation of synonyms) do not affect the status of a name under the International Code of Nomenclature of Prokaryotes. These taxonomic opinions can neither be considered as approved nor as disapproved by the International Committee on Systematics of Prokaryotes.

**Table:** 

Name/authors:	Article no.	Page no.
*Bifidobacterium faecale* Choi *et al.* 2014 pro synon. *Bifidobacterium adolescentis* Reuter 1963 (Approved Lists 1980)	006706	7
*Jeongeupella* corrig. Jiang *et al.* 2024 pro synon. *Antarcticirhabdus* Du *et al.* 2023	006733	6
*Qipengyuania aerophila* Liu *et al.* 2022 pro synon. *Qipengyuania pacifica* Tareen *et al.* 2022	006713	9
*Qipengyuania pacifica* Tareen *et al.* 2022 emend. Lee *et al.* 2025	006713	9
*Saccharopolyspora erythraea* corrig. (Waksman 1923) Labeda 1987 emend. Dif *et al.* 2025	006731	10
*Saccharopolyspora spinosporotrichia* Zhou *et al.* 1998 pro synon. *Saccharopolyspora erythraea* corrig. (Waksman 1923) Labeda 1987	006731	10
*Saccharopolyspora subtropica* Wu *et al.* 2016 pro synon. *Saccharopolyspora thermophila* Lu *et al.* 2001	006731	10
*Saccharopolyspora thermophila* Lu *et al.* 2001 emend. Dif *et al.* 2025	006731	10
*Streptococcus chosunensis* corrig. Lim *et al.* 2024 pro synon. *Streptococcus mitis* Andrewes and Horder 1906 (Approved Lists 1980)*	006704	16
*Streptococcus downii* Martínez-Lamas *et al.* 2020 pro synon. *Streptococcus oralis* subsp. *dentisani* (Camelo-Castillo *et al.* 2014) Jensen *et al.* 2016†	006704	16
*Streptococcus gwangjuensis* corrig. Park *et al.* 2024 pro synon. *Streptococcus mitis* Andrewes and Horder 1906 (Approved Lists 1980)*	006704	16
*Streptococcus hohhotensis* Li *et al.* 2024 pro synon. *Streptococcus mitis* Andrewes and Horder 1906 (Approved Lists 1980)*	006704	16
*Streptococcus humanilactis* Guo *et al.* 2024 pro synon. *Streptococcus mitis* Andrewes and Horder 1906 (Approved Lists 1980)*	006704	16
*Streptococcus mitis* Andrewes and Horder 1906 (Approved Lists 1980) emend. Kilian *et al.* 2025	006704	16
*Streptococcus nakanonensis* corrig. Wajima *et al.* 2025 pro synon. *Streptococcus thalassemiae* Diouf *et al.* 2023	006704	16
*Streptococcus parapneumoniae* Katayama *et al.* 2025 pro synon. *Streptococcus thalassemiae* Diouf *et al.* 2023	006704	16
*Streptococcus toyakuensis* Wajima *et al.* 2022 pro synon. *Streptococcus mitis* Andrewes and Horder 1906 (Approved Lists 1980)*	006704	16

* *Streptococcus mitis* is classified as Risk Group 2.

† *Streptococcus oralis* is classified as Risk Group 2, *Streptococcus oralis* subsp. *dentisani* as Risk Group 1.
